# FPHC Wellbeing Charter: The ‘Whys’ and ‘Hows’ of the Charter

**DOI:** 10.1186/s13049-025-01503-2

**Published:** 2025-11-22

**Authors:** Sarah Morton, Katy Surman, Richard Bayliss, Heather Storey, Emma Gray, Andrew Gant, Alistair Morris, Anna Forbes, Stephanie Cowan, Elizabeth Stevenson, Pamela Hardy, Richard Williams, FPHC Wellbeing Group

**Affiliations:** 1 Imperial College London, Exhibition Road, London, SW7 2AZ UK; 2 FPHC Wellbeing Group, Faculty of Pre-hospital Care, Royal College of Surgeons of Edinburgh, Nicolson Street, Edinburgh, EH8 9DW UK; 3 Barts Health NHS Trust, 9 Prescot Street, London, E1 8PR UK; 4 Welsh Institute for Health and Social Care, University of South Wales, Treforest, Pontypridd, UK

**Keywords:** Wellbeing, Pre-hospital, Psychosocial, Charter

## Abstract

**Background:**

In 2022 the Faculty of Pre-hospital Care (FPHC) report on “Valuing Staff, Valuing Patients” was published, outlining the need to “seek out and remedy secondary stressors”, such as training burdens or financial costs. Since that original publication, COVID-19 and the increased demand for healthcare have presented additional challenges, and staff wellbeing remains an increasing concern. The aim of the FPHC Wellbeing Group was to develop a FPHC Wellbeing Charter, to put the recommendations of the report into practice in a document that outlines achievable measures for all pre-hospital organisations to improve their staff and volunteers’ wellbeing.

**Methods:**

Questionnaires and focus groups, alongside a literature search and the original FPHC report were utilised to develop the Charter. This was led by the FPHC Wellbeing Group. Participants were sought from a range of pre-hospital organisations including National Health Service ambulance trusts, air ambulance organisations and voluntary organisations such as Mountain Rescue. The Charter has been reviewed by the FPHC Executive Committee.

**Results:**

Two hundred eighty-one responses to the questionnaire were obtained and six focus groups were held representing the majority of pre-hospital organisations. As a result of this a FPHC Wellbeing Charter has been developed with four main sections: policies for a good organisation; facilities for a good organisation; support for colleagues in a good organisation and continued professional development, study leave and examination support in a good organisation. Within the policies section there are four sub-sections: rotas and rest; illness/return to work; patient outcome follow-up and parental leave (including maternity policies).

**Conclusion:**

The FPHC Wellbeing Charter outlines ‘why’ and ‘how’ organisations can take measures to improve their staff and volunteer’s wellbeing. Much of the emphasis of the Charter is on reducing secondary stressors by improving simple things, recognising that whilst pre-hospital clinicians and volunteers are often involved in difficult events, daily stresses have a significant cumulative impact. It is anticipated that this will not be a static document; however, a minimum baseline has been set.

## Introduction

In the modern pre-hospital care world staff and volunteers are consistently met with challenges, whether that be intellectual, logistical or emotional. This can take its toll on healthcare staff or volunteers, either due to a particular event, such as in the Manchester Arena Bombing, or with a more cumulative effect, as seen throughout the Covid-19 pandemic or simply by facing the ongoing delays in pre-hospital settings experienced every winter (and often beyond) [1, 2]. Whilst wellbeing has become a buzz word in healthcare, at its heart is the desire to look after oneself and colleagues. Different definitions exist but within this report supporting wellbeing will be defined as “promoting people’s ability to thrive” and psychosocial care as “supporting people who are struggling”. As unique people with different backgrounds and personal situations, reactions and needs can differ from person to person and from time to time.

In 2022 the Faculty of Pre-hospital Care (FPHC) published their report entitled “Valuing Staff, Valuing Patients: the report on the psychosocial care and mental health care programme” [3, 4]. Within this report guidance recommendations were made regarding how organisations can help the wellbeing of staff including the 15 Golden Approaches [3]. As part of these recommendations and Golden Approaches were guidance for pre-hospital organisations such as “seek out and remedy secondary stressors”, adopt a “stepped approach to caring for staff” and “get workplace culture right” [3]. Recommendation seven specially recommended appointing a lead for the FPHC to take this work forward [3, 4]. This was achieved in 2024 with the formation of the FPHC Wellbeing Group, with the Doctor representative on the Faculty Advisory Board as the Lead.

The Wellbeing Group felt that whilst the report outlined many important aspects, further work was needed on more practical suggestions for organisations on how to implement the recommendations. Additionally, it was recognised that representation was needed from all pre-hospital organisations, including the National Health Service, Helicopter Emergency Medical Services and voluntary organisations, and that any changes, particularly following the influence COVID-19 has had on the healthcare system, were incorporated.

Charters exist in a variety of other healthcare systems, for example the British Medical Association (BMA) has both a “Wellbeing” and a “Fatigue and Facilities” charter [5, 6]. Similarly other healthcare organisations have in-house departmental accreditation schemes as a marker of meeting a set of standards, for example the Royal College of Anaesthetists’ ‘Anaesthesia Clinical Services Accreditation’ standards [7]. All of these are publicly available and allow independent review of a healthcare organisation. The aim of the FPHC Wellbeing Group was therefore to develop a FPHC Wellbeing Charter that represented a “good” pre-hospital organisation that considers the wellbeing of their staff and/or volunteers. The aim was for this to be achievable by all pre-hospital organisations, including voluntary organisations, and to normalise good practice; none of the suggestions are intended to have a significant financial cost.

The Charter and this associated report is structured to explain both ‘why’ and ‘how’ all pre-hospital organisations can move forward with implementation of the guidance, to help ensure we value our staff and, as a result, our patients.

## Methods

A qualitative, multi-method design was employed to inform the development of the FPHC Wellbeing Charter. This included a combination of web-based questionnaires, focus groups, a literature search and review of the original FPHC report [3, 4]. This approach allows the integration of stakeholder perspectives with existing evidence to produce a Wellbeing Charter relevant to the pre-hospital care context and the challenges experienced in this setting.

Questionnaire and focus group participants were recruited across a range of pre-hospital care organisations to capture a diverse range of views and experiences. These organisations included NHS ambulance trusts, air ambulance services and voluntary services such as Mountain Rescue. Invitations to participate were distributed via organised mailing lists and social media platforms managed by the FPHC Wellbeing Group. A literature search was also undertaken to identify existing guidance, frameworks and research involving wellbeing in pre-hospital care settings, alongside a detailed review of the FPHC original report to inform the Wellbeing Charter development. Focus groups were conducted with individuals from the same range of organisations, offering an opportunity for collective discussion and elaboration on themes emerging from questionnaire responses. Focus groups were conducted via video conferencing platforms, audio recorded with participant consent and transcribed verbatim. Ethical approval was not required due to the voluntary nature of data collection, as per the National Health Research Authority tool.

Data from the questionnaires and focus groups was thematically analysed using Braun and Clarke’s six-phase framework to identify key themes and priorities [8]. The analysis was conducted by an independent party (KS), external to the FPHC Wellbeing Group to minimise potential bias in interpretation of participant views. Data from the literature review and FPHC report were used alongside these findings to guide development of the Charter. A draft Charter was circulated amongst the FPHC Wellbeing Group and modified until consensus on its content was reached.

## Results

In total, 281 responses were received from the questionnaire (195 male; 86 female) over four months (December 2024-April 2025) and six focus groups were held (January 2025-March 2025), with an average of five participants per focus group. Responses were received from National Health Service (NHS) ambulance service employees (*n* = 78), private ambulance service employees (*n* = 6), Helicopter Emergency Medical Services (HEMS) employees (*n* = 78), British Association for Immediate Care (BASICS) volunteers (*n* = 9), coastguard volunteers (*n* = 1), mountain rescue volunteers (*n* = 91) and other pre-hospital organisation participants (*n* = 18).

The results of the questionnaires and the focus groups were used to develop the Charter described below by the Wellbeing Group. The Charter was also reviewed by the Faculty Advisory Board and their representatives to seek opinion. The final Charter was agreed upon by the FPHC Executive Committee. The Charter has also been endorsed by IBTPHEM (Intercollegiate Board for Training in Pre-Hospital Emergency Medicine).

Specific quotes and comments from the questionnaire were then used as part of the explanatory process below (all anonymised). Whilst not explicitly examined, through the survey responses, focus groups and reflections of the working group, equality, diversity and inclusion have been considered, with recognition that this is an important area in the pre-hospital setting.

### The Charter

**Fig. 1:**
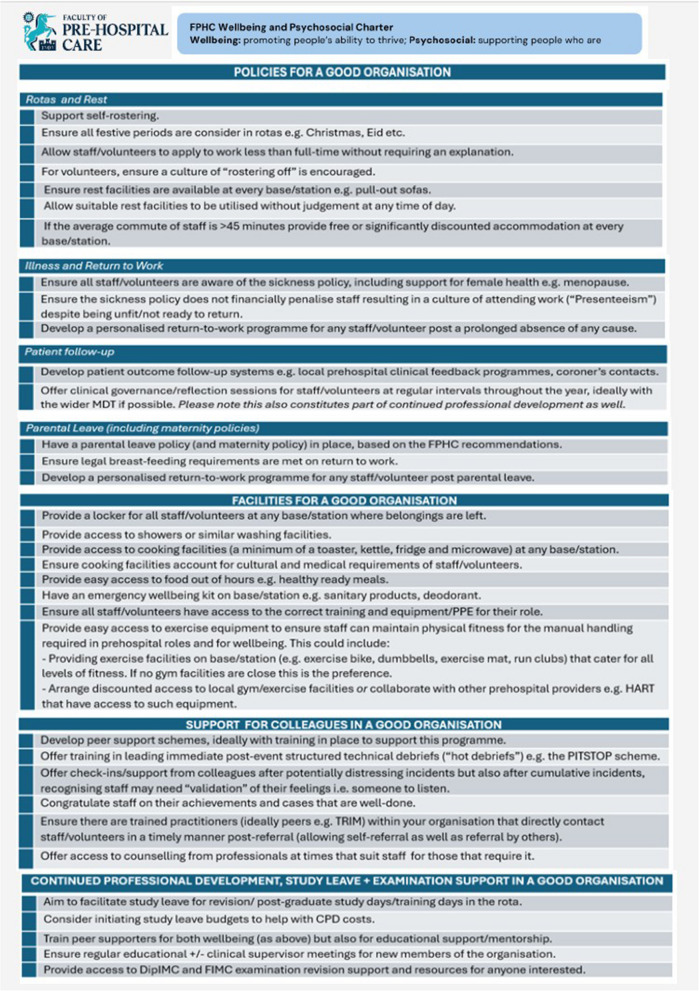
The FPHC Wellbeing and Psychosocial Charter

The Charter is broken down into four main sections: policies for a good organisation; facilities for a good organisation; support for colleagues in a good organisation and continued professional development, study leave and examination support in a good organisation. Within the policies section there are four sub-sections: rotas and rest; illness and return to work; patient follow-up and parental leave (including maternity policies). This is displayed in Fig. 1:

### Policies for a good organisation

#### Rotas and rest

##### Why?

The very nature of pre-hospital care means there is a need for a rapid 24/7 response to patients. However, this has its own implications on the clinicians/volunteers who are required to attend and support these patients. There are known health consequences of working shifts, including the effect on cortisol levels, fatigue and attention [9]. The British Medical Association (BMA) in 2018 published its own charter specifically related to fatigue, accompanied by an associated briefing document [6, 10]. The briefing document carefully summarises the evidence that shift work and fatigue can cause detrimental effects on both staff and patients, for example the increased risk of road traffic collisions seen in night shift workers and the increase in serious medical errors [10]. (Please see the report for more information: https://www.bma.org.uk/media/g1yjtczp/bma-fatigue-sleep-deprivation-briefing-september-2024.pdf).

The effect that rotas and rest can have on wellbeing was echoed throughout the focus groups and questionnaire results. It was unsurprising that respondents commented on the effect shift work can have on health, social interactions and work-life balance, for example “*night shifts place such huge physical demand and cognitive load”*, with the effect on fatigue appearing to increase as age increases. Additionally, outside of volunteer organisations (*n* = 107 respondents), the ability to self-roster was limited (109 out of 174 (63%) non-volunteer respondents could not self-roster). Organisations where self-rostering was the norm were praised, for example “*this is a significant positive and massively impacts on how happy people are at work”.*

The work life-balance is not unique to employees. Volunteers also commented that “*lot of pressure comes from attending calls*” and that they are “*always on call”*. Volunteer organisations that encourage volunteers to “roster-off”, so that they were not always on-call, were praised in the focus groups for reducing the pressure on volunteers and their families and removed some of the guilt volunteers reported when declining a call. It is recognised that these organisations are recruiting sufficient volunteers to facilitate this level of resilience.

In regard to flexible working many felt the opportunity to work less than full time was limited, with 22% of questionnaire respondents stating this was not an option for them. For those who had started to work more flexibly it was noted to have “*improved MH (mental health) and reduced stress”* and “*[I] found it very beneficial”*. For others who did not have this option comments included “*There is however great bias against less than full time staff…”, “this is actively discouraged”* and “*others have requested it [flexible working] … and this has been denied.”* The bias that was described against less than full time staff is of a concern and one that the FPHC wants to eradicate.

Rest facilities were repeatedly highlighted as key. Only 22% of respondents reported rest facilities at their organisation. Whilst it is appreciated that this includes volunteers called from home, the ability to rest after a long call out prior to driving home is important. Organisations which provide rest and sleeping facilities without judgement, and at low to no-cost, were repeatedly praised. Sadly, more commonly reported was an active hostility towards rest, a lack of caring from the organisation or the provision of unsuitable facilities, such as hard chairs or it being very noisy. Several respondents reported comments like “*I wish”, “Haha if only”* or “*the service is very anti sleeping”.* The BMA fatigue and facilities charter outlines the expectation for doctors, and with pre-hospital emergency medicine (PHEM) trainees being part of many pre-hospital organisations, it is not unreasonable to aspire to this for any organisation where clinicians are on-call overnight [6]. The minimum provision should be the provision of suitable pull-out sofas or chairs for resting in breaks.

Inter-linked with rest are the times people commute to their place of work. For many clinicians, being able to achieve a job in pre-hospital care is a career goal and as such there is a willingness to commute. However, over 27% of respondents to the questionnaire were commuting for over an hour, with over 16% often staying away from home to be able to perform their work. This may be influenced by the fixed-term nature of training posts and the overall limited availability of both PHEM training and PHEM consultant posts. The BMA highlights fatigue and sleep deprivation as an area of concern [10]. It is however not unique to doctors. Many studies highlight the danger of lack of sleeping particularly in regards to driving [11–14]. With the risk of traffic collisions in shift workers already being high, such a long commute is of concern, as well as the potential financial implications of accommodation away from home. Others reported having to move to live closer to either their workplace or child-care options due to the significant challenges or arranging childcare around pre-hospital rota hours, potentially causing home upset.

**Table:** 

*Rotas and Rest – How to achieve?*	
- Self-rostering is strongly encouraged. The use of digital or artificial intelligence-based models to perform self-rostering is likely to become more useful as technology improves. Care with how people are rostered must be made, for example using forward-rota designs, as described in the BMA Fatigue and Facilities charter [6].	
- Understand the cultural backgrounds of your staff and ensure all festive periods are considered.	
- Flexible working requests should always be considered, particularly for those returning to work after maternity or parental leave. Answers should be given in a timely manner to ensure sufficient time for any caring responsibilities to be met e.g. arranging childcare, caring for relatives.	
- Actively encourage “rostering off” in volunteer organisations; managers of rotas should lead by example and question if people are continually on call for long periods of time.	
- Ensure suitable facilities exist for rest. The BMA Fatigue and Facilities charter should be used in conjunction with this Charter to achieve this [6].	
- If commutes are > 45 min on average, then accommodation on site should be offered between shifts. If this is not possible, significantly discounted accommodation should be offered in the very local vicinity, to minimise the risk of driving. Organisations should work with local accommodation to achieve this. It is hoped with time that organisations will be able to offer accommodation for any clinician requiring this between shifts, as per the BMA recommendations [10], and the limit is lowered to a > 30 min commute	

#### Illness and return to work

##### Why?

Illness and injury will occur in any line of work, but there are additional implications of working in the pre-hospital environment which is associated with an increased injury burden [15]. In the wider NHS there is a recognition that staff sickness since the COVID-19 pandemic has increased [16]. 24% of staff/volunteers stated that they were not aware of a sickness policy within their own organisation, despite a requirement on employers to provide this [17]. 54% stated however that they were aware of a return to work programme, although comments highlighted a lack of clarity: “*it is not official enough…there should be an equitable agreed process”*, *“it was very ad-hoc”* and “*if the return to work had been managed properly, I would be back on my shift now”*. Of the 70 survey respondents who had experienced a return to work, 40 reported a negative experience with minimal support. In those organisations where clear return to work policies have been implemented, organisations were praised, for example “*very supportive following injury with phased return”* and *“well supported with alternate non frontline duties while recovering from injury”*. Again, it is a statutory requirement to ensure that workplace adjustments are made to help people return to work [17], and its importance is reflected by its inclusion in the BMA Mental Wellbeing Charter [5].

However, alongside this is the concern regarding “presenteeism”, whereby staff or volunteers attend work despite not being at full health. Presenteeism has been shown to be associated with an increased rate of near-misses within paramedics [18], and has been highlighted as a concern in a recent independent culture review of ambulance trusts commissioned by the NHS [19]. There was a lot of concern about the financial implications of taking time off, for example “*I feel financially punished for sickness”,* “*I only get paid if I turn up to shift”* and *“people are afraid of going sick and therefore coming into work when they shouldn’t”*. Additionally, there still exists stigma and difficulty around mental health concerns, for example “*still a stigma around taking time off for mental health issues”*.

Aside from the need for a parental leave policy, as described below, there is also a need to consider female health issues. It is estimated that 80% of women experience period pain [20], and the menopause impacts many women, including an estimated 10% of women who leave their jobs due to menopausal symptoms [21]. This is an area that is likely to require increased consideration and research in the near future.

**Table:** 

*Illness and Return to Work – How to achieve?*	
- Ensure every pre-hospital organisation has an illness policy in line with the Health and Safety Executive requirements [17].	
- Ensure the culture of “presentism” is removed, including ensuring there are no significant financial implications to staff.	
- Adapt return to work policies to cater to the differing environments and requirements of both clinical and non-clinical staff to prevent people returning to clinical duties too early.	
- Consider female health and the implications this may have on this sub-set of employees/volunteers, for example around the menopause and period health. This is an area that is likely to require research in the near future.	
Ensure return to work programmes are individualised, based on the reason for a period of absence and the individual’s own background/experience. Consider flexible working and temporary redeployment to non-front-line duties as part of this.	

#### Patient outcome follow-up

##### Why?

Patient follow-up is important for both individual and system level learning as well as supporting the wellbeing of clinicians. When formal feedback is available from secondary care most clinicians suggested it would be likely to change their clinical practice and most clinicians found it positively impacted their mental health [22]. This was reflected in focus groups as well as published literature. Follow-up may be immediate or long term. Sources of information for follow-up generally include secondary care clinicians and records, Major Trauma Networks or where a patient has died, information may arise from the coronial service e.g. post-mortem reports. Pre-hospital critical care organisations may have aftercare services that include links to specific hospitals as well as allowing contact from patients and their families or support network which may provide more nuanced and longer term follow-up [23].

The main barriers to effective feedback are resource constraints and perceived information governance issues [24]. This is despite the General Medical Council statement that “good medical professionals…reflect regularly…and use feedback…to develop personal and professional insight” [25]. Information from coronial inquiry may not be available for considerable time following an incident due to the requirement for legal processes to be completed prior to its release. Frequently pre-hospital clinicians circumvent these barriers utilising informal mechanisms [24], which presents professional and organisational risk. It has been recognised at a government level that this reflective practice is integral to safe care, with 97% of the general population polled believing it was important that healthcare professionals had access to patient follow up information [26]. Through the use of formalised processes and information sharing agreements between organisations feedback can be delivered in a way that upholds good information governance whilst improving the service that patients receive and enhancing the well-being of the clinicians who have been involved in that patients care.

**Table:** 

*Patient outcome follow up – How to achieve?*	
- Regular clinical governance sessions with senior clinicians are recommended to support reflective practice. The purpose of these sessions as an educational tool for personal and organisational learning should be emphasised and cases with excellent practice should be reviewed as well as those where there are opportunities for improvement.	
- Organisations should adopt a structured technical debrief model to maximise consistency for participants and encourage psychological safety, differing from reflections of the emotions of the case. HEMS organisations may be able to offer support to other organisations for suitable structures in settings such as Morbidity and Mortality meetings (or similar).	
- Pre-hospital care providers should develop information sharing agreements with local clinical networks from whom they are referred patients and to whom they hand over the care of patients.	
- The Faculty of Pre-Hospital Care supports the clinical, legal and moral basis for the provision of feedback including patient clinical information to pre-hospital care providers for the purpose of clinician reflective practice, service development and psychological well-being.	
- Organisations should endeavour to develop links with local coronial and pathologist services and develop mechanisms to routinely receive reports from death investigations where patients have been treated by their clinicians. Services are encouraged to collate objective and accurate data regarding the outcomes of their patients.	

#### Pregnancy and parental leave policy

##### Why?

Throughout the focus groups and questionnaire responses it was clear that the approach to pregnancy, or lack of approach, has resulted in unnecessary stress and a huge impact on wellbeing. Seventeen percent of respondents stated that their pre-hospital organisation did not have a pregnancy policy (mainly HEMS organisations and some voluntary sectors), with an additional 30% not knowing if there was one. Several individuals reported knowing of colleagues who had concealed pregnancies for as long as possible. Quotes from the questionnaire included: “*I have heard of people concealing pregnancies…*”, “*Female colleagues suffer the detrimental effect of being removed from operation duties as soon as they make their pregnancy public”* and “*I have sadly worked in organisation where someone appeared to be dismissed because of their pregnancy”*. It was highlighted in both the focus groups and questionnaire results that employees on emeritus, contractor or zero-hour contracts were particularly affected. Paternity leave was also recognised to be limited. Additionally, it was recognised that breastfeeding support was inadequate in many places, with a lack of space to store milk or to express breast milk in a suitable space on returning to work; toilets are not suitable spaces. Regarding returning to work quotes included “*colleagues returning to practice following maternity leave suffer due to challenges trying to sort out shifts, lack of planning…and significant periods away from clinical exposure”.* Sadly, women reported feeling overall “*less respected”* and “*punished”.* Organisations where return to work planning was performed on a more individualised basis were congratulated; however, this did not appear to be normal practice.

Pregnancy is a protected characteristic under the Equality Act 2010 [27]. Additionally legislation exists around maternity and parental leave [28]. There is also guidance from the Health and Safety Executive around protecting pregnant workers and new mothers [29]. It is of course appreciated that the complex and physical nature of pre-hospital work is challenging but it is important that pregnant women are afforded informed choice [30]. As such the FPHC has, as part of this wellbeing work and to complement the Charter, developed a separate document entitled “FPHC parental leave guidance” [31]. Within this document the existing literature is reviewed in more detail, the legal requirements outlined and a suggested policy described, including considerations on return to work [31].

**Table:** 

*Pregnancy and parental leave policy – How to achieve?*	
- Ensure that the organisation’s parental and maternity leave policy meets the recommendations outlined in the separate FPHC parental leave guidance [31]. A key aspect of this is informed choice and a personalised risk assessment.	
- All legal obligations as outlined in the Equality Act and maternity legislation must be met.	
- Make sure the parental leave and maternity policy is easily accessible to all staff/volunteers so that they can have full understanding of what they can expect.	
- Support breastfeeding, for example with a fridge for storage or dedicated suitable spaces, as per the HSE guidance [29].	

#### Facilities for a good organisation

##### Why?

Pre-hospital care encompasses the whole of the United Kingdom, and to facilitate this operational bases/stations often exist in relatively isolated places. Unlike in hospitals, which are often sited in major towns or cities with either access to local shops or delivery services, pre-hospital bases may not. Consequently, the access to facilities may be limited and therefore these need to be provided on base.

It is known that insufficient sleep will increase the drive to eat [32]. On top of this increase drive to eat, due to the nature of shift work, people eat late at night and often poor quality food [33]. This in turn has meant shift work has been associated with an increased risk of developing obesity [33]. Diet has therefore been recognised a key theme in wellbeing strategies [34]. Various editorials exist advising what should be eaten on night shifts [35]. However, access to “healthy food” may be limited; multiple respondents would like to see “*healthy meals”* be available. The BMA Fatigue and Facilities charter echoes this sentiment, stating that there should be a catering facility open 365 days a year providing varied and freshly prepared meals [6]. Organisations may however operate with relatively few employees and it is appreciated this has a cost implication. Practical suggestions could therefore include vending machines with healthy meal options (minimally processed), as many hospitals have utilised to meet the BMA recommendations, or a small number of emergency frozen meals that are checked weekly to ensure sufficient are available with an honesty box for those who utilise them. Care must be taken to avoid ultra-processed foods as there is increasing evidence that this has its own implications on health [36–38]. As noted below, the access to refrigerated and non-refrigerated space is likely to be key to allow people to bring in their own food, avoiding ultra-processed ready meals. Similarly, due to the difficulty of “going to the shops” associated with working on pre-hospital bases, all organisations should have access to a simple emergency wellbeing kit that provides immediate access to products such as sanitary products. Its estimated that 86% of women have started their period at work without having the necessary products available [39]. These are secondary stressors that can result in embarrassment but can be avoided.

Alongside access to food is storage and preparation of food. Several respondents stated that the lack of fridge on their base meant it difficult to bring in food. Others reported that medical and cultural requirements provided difficulties in storing and preparing food, for example cross-contamination with gluten for staff with Coeliac disease. In-hospital there is an expectation of having a “common room” or “mess” that provides suitable facilities [6]. This might not all be feasible in a smaller pre-hospital organisation but a minimum set of cooking facilities should be provided at every base/station, with considerations made to include all colleagues.

Pre-hospital clinicians and volunteers work in environments that are more extreme than in-hospital, often being dirty and resulting in exposure to bodily fluids. To be expected to then get in your car to go home with blood or dirt on oneself should not be considered appropriate by Infection, Prevention and Control polices and is an additional stress highlighted in the responses, with a request for showers on base being made repeatedly. Again these facilities are highlighted as important in the BMA Fatigue and Facilities charter, and have been described before as a simple win for staff morale and welfare [6, 40]. They also provide clinicians/volunteers the ability to exercise and shower around their work hours, or, for example, cycle to work. Similarly, ensuring there is a personal locker to keep valuables safe during work time is crucial [40].

Exercise, including walking, was one of the most frequently mentioned strategies for improving wellbeing in both the focus group and questionnaire responses. 75% of respondents use exercise as method to support their wellbeing, second only to support from peers (91%). However, only 19% of respondents had exercise equipment available at work. This is despite both the wellbeing and physical health benefits that exercise has. This finding fits with similar studies in clinicians [34]. In a free text question when asked “If money was no object what would you like to see implemented for your wellbeing”, 20% of respondents wrote they would like access to exercise facilities on base. Organisations where exercise facilities were available were repeatedly praised. The literature suggests that, for example, regular fitness classes will decrease stress and improve physical, mental and emotional quality of life [41]. They are also likely to have a beneficial effect on preventing musculoskeletal disorders and injuries that occur commonly in the pre-hospital environment. The annual prevalence in emergency medical technicians and paramedics of back pain is estimated to range between 30–88%, having a huge impact on sick leave [42]. Musculoskeletal problems have long been recognised as a leading cause for sickness absence [43]. It should not be forgotten that as part of the application process to become a PHEM trainee, and for many other pre-hospital organisations, a fitness test is required and there should be an expectation and duty of care to ensure this level of fitness is, and can be maintained [44]. Therefore, access to exercise equipment is key not only in order to be able to perform our jobs but also to prevent injury.

Finally, it is a legal requirement that all staff/volunteers have access to suitable Personal and Protective Equipment (PPE), including daily uniform requisite to the role [45]. The Health and Safety Executive (HSE) states PPE must be provided, compatible, maintained, correctly stored and used properly; alongside this training and instruction must be provided to workers [45]. The lack of PPE provided to healthcare workers during the Covid-19 pandemic had a huge impact on front-line staff, resulting in anxiety and distress [46]. However, it extends beyond, for example, masks and includes flight helmets and boots. This continues to be of concern, with respondents to the questionnaire highlighting the lack of access to “*functional and fit for purpose equipment*”, with uniform also an area that respondents felt was not always suitable.

**Table:** 

*Facilities for a good organisation – How to achieve?*	
- Ensure staff/volunteers have access to healthy food options 24/7. Practical suggestions could include vending machines with healthy meal options (avoiding ultra-processed food) or a small number of emergency frozen meals that are checked weekly to ensure sufficient are available with an honesty box for those who utilise them. Frozen meals should include vegetarian, vegan and Coeliac-friendly options to cater for most major cultural and medical conditions. Sufficient space to store food safely should be provided.	
- Ensure staff/volunteers have access to a bare minimum of cooking facilities. These can be small, and donations could be sought from local businesses to cover any costs of providing them. More information on this can be found on the BMA Fatigue and Facilities charter [6].	
- Cultural and medical requirements should be considered with catering facilities. For example, a shelf at the top of the fridge should be “meat free” and in a 4-rack toaster one rack should be kept as gluten free only.	
- Provide an emergency wellbeing kit on base including as a minimum sanitary product. Again, an honesty box could be provided to ensure this is restocked on a weekly basis.	
- PPE must be provided in line with HSE regulations. Staff/volunteers should be regularly audited anonymously to ensure they are happy with the quality of the equipment and that training in its use is sufficient.	
Ensure easy access to exercise equipment on base, ideally 24/7, so that staff can benefit from both the physical fitness and mental health benefits exercise brings. As for cooking facilities, donations could be sought from local businesses for simple equipment that does not require much space to be available on base, such as exercise bikes and dumbbells. Additionally, discounts with local gym organisations could be arranged, for example working with organisations such as Blue Light Card to facilitate this for employees/volunteers who may not have an NHS email address. Sharing of resources may also be possible, e.g. working with the Hazardous Area Response Team (HART).	

#### Support for colleagues in a good organisation

##### Why?

As was outlined in the 2022 publication, it is important that everyone’s wellbeing is nurtured, promoting their ability to thrive [4]. There is a spectrum; psychosocial care encompasses the needs of people who are struggling, but are likely to recover with the support of friends and family and without the need for medical intervention [4]. There is then a small number of people who have a diagnosable mental health disorder and require specialist assessment and treatment [4]. Unfortunately, many of us will know pre-hospital practitioners who have sadly died due to suicide, and whilst this may not be directly attributable to work, it is imperative on us to try our best to recognise this spectrum and to try and prevent the progression.

A lot of what is described in the Charter relates to secondary stressors and how we can limit them. However, it is recognised that people working in the pre-hospital environment will be involved in the care of people in distressing situations resulting in primary stressors [4]. Whilst we cannot prevent this as it is intrinsic to pre-hospital work, the aim is to support each other and colleagues. Support from peers was the most important mechanism people identified for supporting their wellbeing, with 91% selecting it as a coping method, above even family and friends. During focus group discussions, it was evident that peer support from colleagues was highly valued due to the insight into what participants had experienced and additionally were able to offer easily accessible informal debriefs. Indeed, terms such as “*work spouse”* were used to show the significance of these relationships for some. What was clear, is the importance of finding the right person for that relationship – enforced mentoring appeared less successful. There is a growing body of evidence that within healthcare, that peer support is of benefit and it can help provide an efficient recovery from a traumatic event as well as giving support to the professional involved, and is an area of pre-hospital medicine that requires additional research moving forward [47].

Many people described wanting to feel listened to (i.e. validated) but without constant checking-in; one person described “*the second hit of finishing a difficult or distressing mission and then being asked over several days, *via* several routes…are you ok?”* and another “*you can even think it’s intrusive that someone’s messaging you all the time and actually I’m fine”*. Care therefore must be taken to balance this. Also, people worried *“with everyone asking after my wellbeing, does it mean I lack empathy…by NOT being emotionally crippled every time…”.* Others commented that it is the cumulative effect of jobs that can take its toll, rather than a single job per se: *“it can be very difficult to define what job is going to be a trigger for somebody”.* This should be remembered, and it may be that a check-in after cumulative incidents is of equal, or more, value than simply what one person defines as a “big job”.

Alongside informal support schemes respondents did suggest there are occasions where more formal support is required. Trauma Risk Management (TRIM) practitioners were often mentioned as they are more in line with the peer group support and many found them positive. However, difficulties described included concern about confidentiality, lack of utilisation and timeliness being a problem. This was echoed in the focus group as people described experiences of formal counselling only being offered Monday to Friday 9am-5 pm at a set time of the week, incompatible with the nature of pre-hospital rotas. As such, charities such as the Benevolent Trust and Lifelines Scotland were instead valued more. This is echoed in the BMA Mental Wellbeing Charter, which emphasises making support services accessible [5]. One focus group described how in their service peer support had helped colleagues make the first phone call to more formal support organisations/counselling, described as “bridging”, as this was felt to be the hardest call to make and this may be of benefit to other organisations to consider.

Respondents also felt that often negatives were concentrated on, and staff were rarely praised for doing something well. Paediatricians have led the way on developing positive feedback systems such as “Greatix” and such systems have been shown to boost staff morale if implemented in the correct way [48, 49].

Pre-hospital care often brings together flash teams working for several different organisations at high acuity events. This may include teams from other emergency services, private organisations and charities who all contribute to the acute phase of patient care; these teams may struggle to reconnect in the same way again due to time constraints and different rotas. Focus group participants found value in “hot” debriefs, i.e. immediately at the time of the event, to ensure everyone was involved and also stated that “cold” debriefing at a later stage was useful but occurs less frequently due to the problems of reuniting the team described above. Of the survey respondents 60% reported debriefs and case reviews as helpful methods to support their wellbeing. However, there are significant concerns that “hot” debriefs at the time of the event can cause harm, especially when they attempt to offer feedback at a time when clinicians are often not ready for it, or attempt a psychological debrief, which requires additional training. The literature supports that a structured technical debrief can have a beneficial effect on participants, especially when those facilitating sessions have had training [50]. As a result the recommendation should be on a structured technical debrief, aiming to ensure that no member of the flash team leaves with questions about the timeline of events or a mis-understanding around the decision making involved that can later result in psychological harm. Tools already exist for this and should be encouraged, such as “PITSTOP” (Pause, Intention, Talk through ground rules, Summarise, Thank & acknowledge, Opportunity for reflection and Promote wellbeing) and “STOP5” (Summarise, Things that went well, Opportunities to improve and Points to action) [51, 52]. The aim of this debrief should be set out clearly from the outset, e.g. summarising the case, outlining immediate concerns (e.g. this piece of equipment was broken and needs to be removed from service) and signposting people to additional support services if required. However, nobody should be forced to attend a debrief as this is likely to have a negative impact in the long term. Additionally, people should not be asked or expected to discuss the emotional impact of a case at a technical debrief, unless this occurs naturally and in a comfortable way. “Cold debriefs”, which may be incorporated into routine case review meetings, provide opportunities to maximise individual and organisational learning as well as protecting the well-being and psychological health of clinicians within a system at a later stage [53]. They can form part of broader governance and quality improvement.

**Table:** 

*Support for Colleagues in a Good Organisation – How to achieve?*	
- Encourage peer support schemes. As detailed further below training may be beneficial, but also ad hoc mentoring that develops may be as useful and should be valued by organisations.	
- Check-ins can be very worthwhile but repeated check-ins about the same job need to be kept to a minimum as it may have detrimental effect. Cumulative jobs should also be considered when checking-in.	
- Develop systems to congratulate staff/volunteers on jobs done well. This can include systems such as “Greatix” or feedback in governance sessions.	
- Ensure easy access to information to employees/volunteers on what help is available, where it is available from and how to access the different types as needs arise.	
- Ensure any formalised counselling/professional support is offered at times that work for employees/volunteers. Facilitating that first phone call (or similar) may be of benefit i.e. bridging them to the support rather than just signposting. If as an organisation formalised counselling etc. cannot be offered in-house ensure easy access on the intranet, newsletters or similar to relevant charities than can support employees/volunteers.	
- If utilising TRIM or similar systems, ensure there is a system in place to firstly allow easy referral (either self-referral or by others) and follow-up is made in a timely manner. Recognise that clinicians and volunteers may need this support after a particular event, or in certain roles where exposure is more common (for example HEMS or Hazardous Area Response Teams (HART)) after a period of time rather than one particular event.	
- Offer training in how to run an immediate (“hot”) structured technical debrief, ideally following a structure that exists within the literature and can be easily repeated. It should focus on the facts about what have occurred and should not be a psychological debrief.	
- Consider pre-existing charters, such as the BMA mental wellbeing charter and the United Kingdom Search and Rescue guiding principles for emergency response volunteer wellbeing to provide additional suggestions alongside this Charter to aid implementation [5, 54].	

#### Continued Professional Development (CPD), study leave and examination support in a good organisation

##### Why?

Pre-hospital medicine is an ever-evolving speciality. It is only since 2011 that PHEM has been recognised as sub-speciality, with trainees entering training since August 2014 [55]. All pre-hospital practitioners and volunteers will be expected to maintain their level of competence [56], and many will want to advance their training, often in line with the FPHC descriptors [57]. Respondents throughout the questionnaire and focus groups recognised the importance of ongoing education.

Over 61% of respondents reported studying for exams, qualifications or university degrees alongside their (often full-time) pre-hospital work. Many have portfolio careers, with parallel commitments and exams. Competition to obtain pre-hospital jobs often drives this ongoing studying, with one respondent reporting “*it’s almost expected that you’re undertaking a qualification within pre-hospital care”.* However, study time was limited, as people were often doing this alongside full-time jobs, with comments including “[it is] *very stressful in combination with work and family”* and “*governance days, training days *etc*. sessions are done outside of work time and people are viewed as less than if they don’t attend”*. Respondents also commented on the additional significant financial costs that they have incurred, whether this be by travelling for any examination or studying for university degrees.

Support, in terms of revision material or study leave, for studying for pre-hospital examinations was also described as limited; PHEM training in particular was highlighted as having a “*high exam burden”*, as was described in the initial FPHC report [4], with a “*lack of resources”*, sometimes “*behind paywalls* “ (i.e. resources required had a financial cost to obtain a copy) and significantly fewer resources than “*equivalent in-hospital exams”*. The Wellbeing Group recognise that PHEM remains a relatively new sub-specialty, with examinations mostly developed by volunteers and therefore, as many of these volunteers are active clinicians themselves, there is a balance here. However, amongst the focus groups it was clear that there is a discrepancy across the UK about the support some people receive, both financially and in terms of revision. Some PHEM trainees highlighted that they received the benefit of “*mandatory study days and study leave allowance and a study budget”* but did comment that those outside of this system, who want to do the same examinations, do not receive the same. The psychological impact of failing examinations was also highlighted, particularly due to the relatively small number of people sitting some of the examinations. This is not a surprise, with the existing literature showing higher levels of psychological distress in those who fail examinations [58]. Certain areas of the country were praised for implementing programmes to try and help those starting to revise for exams. As such, study leave should be considered on a case-by-case basis, including independent study (rather than for a particular course). Additionally, there should be resource sharing across the UK for those studying for the FPHC examinations, with easy access to such resources for all. It is hoped with time this begins to replicate and expand the resources and courses that exist within other colleges, such as videos with mock stations, example questions and course provided by the Faculty of Intensive Care Medicine [59].

One of the recommendations from the initial FPHC “Valuing Staff, Valuing Patients” report was the development of peer support programmes [3, 4]. This is likely to have a benefit on wellbeing, as described above, but also may help with specific educational needs and questions. The Intercollegiate Board for Training in Pre-Hospital Emergency Medicine (IBTPHEM) was suggested in the original report as a starting point for the development of peer support, but it is likely to have wider benefit than for PHEM trainees alone [60]. The Intensive Care Society have adapted a similar strategy and found this to be beneficial, particularly during the Covid-19 pandemic [61, 62].

Associated with this, yet separate, is ensuring practitioners have access to suitable clinical and/or educational supervision. This allows people the benefit of developing their own educational goals or personal development programmes, focussing on their own learning. All PHEM trainees are expected to have regular meetings to ensure educational learning goals are being met, but there are benefits for others, as well as requirements for ongoing revalidation [63]. Outside of these more formal requirements, it will also allow for new members of any pre-hospital organisation to gain some initial supervision and, hopefully, gain insight from the pre-existing knowledge base within an organisation.

Although discussed separately above, it should also not be forgotten that patient outcome follow-up is part of the continued professional development of a practitioner.

**Table:** 

*Continued Professional Development (CPD), Study leave and Examination Support in a Good Organisation – How to achieve?*	
- Consider study leave on a case-by-case basis and this can include independent study days, rather than for a specific course. Around the time of, for example, examinations or dissertation write-ups care should be given to factoring this into rota planning, so people are not on nights the day prior to deadlines.	
- Ensure support for specialist training and certification requirements, for example the necessary qualifications for those working in HART or in an advanced paramedic role.	
- Encourage the sharing of study resources, particularly for examination revision, within an organisation and to the wider pre-hospital community as interested.	
- Study leave budgets should be encouraged for all to apply for within an organisation, not only PHEM trainees. Each application can then be reviewed on its own merit.	
- Organisations should be encouraged to pool their resources regarding revision support. For example, if a revision course is being run, online access could be given for others to join across the country to provide some equity of access.	
- Develop peer support training schemes, in line with the original suggestions from the FPHC. The FPHC is currently working to develop a training package to facilitate, similar to the Faculty of Intensive Care Medicine program developed by Professor Richard Williams.	
- Provide educational and/or clinical supervisors for any new member of a pre-hospital organisation.	

## Conclusion

The Faculty of Pre-hospital Care Wellbeing Charter outlines achievable measures for pre-hospital organisations to improve their staff and volunteers’ wellbeing. Much of the emphasis of the Wellbeing Charter is on reducing secondary stressors by improving simple things, recognising that whilst pre-hospital clinicians and volunteers are often involved in difficult events, daily stresses have a significant cumulative impact. By improving secondary stressors as suggested in this document, it is hoped that wellbeing will be improved, and these considerations will become normal. It is also anticipated that this will not be a static document, and that with time and changes in the pre-hospital setting, this is likely to require updating. Alongside this all pre-hospital organisations are encouraged to continue to review any health and wellbeing support implemented to ensure it remains relevant and up-to-date, actively involving staff and volunteers in this process. However, the FPHC believes a minimum baseline has been set and encourages all relevant organisations to sign up to it in a commitment to “Valuing Staff, Valuing Patients”.

## Data Availability

No datasets were generated or analysed during the current study.

## References

[1] Murray E, Kaufman KR, Williams R. Let us do better: learning lessons for recovery of healthcare professionals during and after COVID-19. BJPsych Open. 2021;7:e151. 10.1192/bjo.2021.981. 34457351 10.1192/bjo.2021.981 PMC8376907

[2] Stancombe J, Williams R, Drury J, Collins H, Lagan L, Barrett A, et al. People’s experiences of distress and psychosocial care following a terrorist attack: interviews with survivors of the Manchester Arena bombing in 2017. BJPsych Open. 2022;8:e41. 10.1192/bjo.2022.2. 35109959 10.1192/bjo.2022.2 PMC8867861

[3] FPHC The Royal College of Surgeons Edinburgh. Valuing Staff, Valuing Patients: The report on the psychosocial care and mental health programme. 2022. https://fphc.rcsed.ac.uk/media/3140/valuing-staff-valuing-patients.pdf. Accessed 8 Aug 2025.

[4] Williams R, Kemp V, Burgess J, Murray E, Stokes S, Wood A, et al. Practical psychosocial care for providers of pre-hospital care: a summary of the report “valuing staff, valuing patients.” Scand J Trauma Resusc Emerg Med. 2023;31:77. 10.1186/s13049-023-01141-6. 37946286 10.1186/s13049-023-01141-6 PMC10636848

[5] British Medical Association. BMA mental wellbeing charter. 2024. https://www.bma.org.uk/media/mqql3veu/bma-mental-wellbeing-charter-september-2024.pdf. Accessed 30 April 2025.

[6] British Medical Association. BMA Fatigue and Facilities charter. 2018. https://www.bma.org.uk/media/juxn0f5i/bma-fatigue-and-facilities-charter-september-2024.pdf. Accessed 30 April 2025.

[7] Royal College of Anaesthetists. The ACSA standards. 2025. https://www.rcoa.ac.uk/safety-standards-quality/anaesthesia-clinical-services-accreditation/acsa-standards. Accessed 30 April 2025.

[8] Braun V, And CV. Using thematic analysis in psychology. Qual Res Psychol. 2006;3:77–101. 10.1191/1478088706qp063oa.

[9] Niu SF, Chung MH, Chen CH, Hegney D, O’brien A, Chou KR. The effect of shift rotation on employee cortisol profile, sleep quality, fatigue, and attention level: a systematic review. J Nurs Res. 2011;19:68–81. 10.1097/JNR.0b013e31820c1879. 21350389 10.1097/JNR.0b013e31820c1879

[10] British Medical Association. Fatigue and sleep deprivation - the impact of different working patterns on doctors. 2018. https://www.bma.org.uk/media/g1yjtczp/bma-fatigue-sleep-deprivation-briefing-september-2024.pdf. Accessed 30 April 2025.

[11] Huffmyer JL, Moncrief M, Tashjian JA, Kleiman AM, Scalzo DC, Cox DJ, et al. Driving performance of residents after six consecutive overnight work shifts. Anesthesiology. 2016;124:1396–403. 10.1097/aln.0000000000001104. 27028468 10.1097/ALN.0000000000001104

[12] Williamson AM, Feyer AM. Moderate sleep deprivation produces impairments in cognitive and motor performance equivalent to legally prescribed levels of alcohol intoxication. Occup Environ Med. 2000;57:649–55. 10.1136/oem.57.10.649. 10984335 10.1136/oem.57.10.649 PMC1739867

[13] Tefft BC. Acute sleep deprivation and culpable motor vehicle crash involvement. Sleep. 2018;41. 10.1093/sleep/zsy144. 10.1093/sleep/zsy144 30239905

[14] Barger LK, Cade BE, Ayas NT, Cronin JW, Rosner B, Speizer FE, et al. Extended work shifts and the risk of motor vehicle crashes among interns. N Engl J Med. 2005;352:125–34. 10.1056/NEJMoa041401. 15647575 10.1056/NEJMoa041401

[15] Kearney J, Muir C, Smith K. Occupational injury among paramedics: a systematic review. Inj Prev. 2022;28:175–84. 10.1136/injuryprev-2021-044405. 34972683 10.1136/injuryprev-2021-044405

[16] Nuffield Trust. NHS in England grappling with difficult new normal as staff sickness soars post-panedmic. 2023. https://www.nuffieldtrust.org.uk/resource/all-is-not-well-sickness-absence-in-the-nhs-in-england. Accessed 8 May 2025

[17] Health and Safety Executive. Managing sick leave and return to work. 2025. https://www.hse.gov.uk/sicknessabsence/. Accessed 8 May 2025.

[18] Ishimaru T, Kubo T, Honno K, Toyokuni Y, Fujino Y. Near misses and presenteeism among paramedics. Occup Med. 2019;69:593–7. 10.1093/occmed/kqz076. 10.1093/occmed/kqz076 31206581

[19] NHS England. Culture review of ambulance trusts. 2024. https://www.england.nhs.uk/long-read/culture-review-of-ambulance-trusts/. Accessed 8 May 2025.

[20] Women's Health Concern. Information for women: period pain. 2022. https://www.womens-health-concern.org/wp-content/uploads/2022/12/20-WHC-FACTSHEET-Period-Pain-NOV2022-B.pdf. Accessed 8 May 2025.

[21] Newsome L. Women's experiences of perimenopause and menopause. 2024. https://www.newsonhealth.co.uk/wp-content/uploads/2024/10/DLN-Menopause-Consumer-Research-April-24-FINAL-Oct1.pdf. Accessed 8 May 2025.

[22] Snowsill M, Cracolici G, Wieder T, Allen G. Facilitated hospital-to-pre-hospital feedback for professional development (PHEM feedback): a service evaluation using a self-reported questionnaire to understand the experiences of participating pre-hospital clinicians in the first year of operation. Br Paramed J. 2023;8:42–52. 10.29045/14784726.2023.6.8.1.42. 37284605 10.29045/14784726.2023.6.8.1.42 PMC10240859

[23] Plumbley S, Taneja S, Griggs J, Al Rais A, Curtis L, Lyon R. Patient and family aftercare enhance interactions between helicopter emergency medicine services and former patients and families. BMC Health Serv Res. 2024;24:1238. 10.1186/s12913-024-11720-7. 39407209 10.1186/s12913-024-11720-7 PMC11481800

[24] Eaton-Williams P, Mold F, Magnusson C. Exploring paramedic perceptions of feedback using a phenomenological approach. Br Paramed J. 2020;5:7–14. 10.29045/14784726.2020.06.5.1.7. 33456380 10.29045/14784726.2020.06.5.1.7 PMC7783907

[25] General Medical Council. Good medical practice. 2025. https://www.gmc-uk.org/professional-standards/the-professional-standards/good-medical-practice. Accessed 30 April 2025.

[26] Byrne N. National Data Guardian 2023–2024 report: Section 5.15. 2024. https://www.gov.uk/government/publications/national-data-guardian-2023-2024-report/national-data-guardian-2023-2024-report#supporting-the-development-of-data-policy-and-guidance. Accessed 30 April 2025.

[27] UK Government. Equality Act 2010: guidance. 2010. https://www.gov.uk/guidance/equality-act-2010-guidance. Accessed 30 April 2025.

[28] UK Government. The Maternity and Parental Leave etc. Regulations 1999. 1999. https://www.legislation.gov.uk/uksi/1999/3312/contents. Accessed 30 April 2025.

[29] HSE. Protecting pregnant workers and new mothers: employers: 4. Rest and breastfeeding at work. 2025. https://www.hse.gov.uk/mothers/employer/rest-breastfeeding-at-work.htm. Accessed 8 June 2025.

[30] Storey HM, Austin J, Davies-White NL, Ransley DG, Hodkinson PD. Navigating pregnancy for employees in civilian rotary-wing aeromedicine. Aerosp Med Hum Perform. 2022;93:866–76. 10.3357/amhp.6115.2022. 36757253 10.3357/AMHP.6115.2022

[31] Storey HM, Stokes S, Surman K, Hardy P, Morton S et al. FPHC Wellbeing: Pregnancy, maternity and return to work guidelines 2025. https://fphc.rcsed.ac.uk/education-resources/resources/fphc-psychosocial-project-working-group. Accessed 10 Nov 2025. 10.29045/14784726.2026.9.11.2.64 PMC13532032 42683383

[32] Chaput JP, Mchill AW, Cox RC, Broussard JL, Dutil C, Da Costa BGG, et al. The role of insufficient sleep and circadian misalignment in obesity. Nat Rev Endocrinol. 2023;19:82–97. 10.1038/s41574-022-00747-7. 36280789 10.1038/s41574-022-00747-7 PMC9590398

[33] Marot LP, Lopes T, Balieiro LCT, Crispim CA, Moreno CRC. Impact of nighttime food consumption and feasibility of fasting during night work: a narrative review. Nutrients. 2023. 10.3390/nu15112570. 37299533 10.3390/nu15112570 PMC10255296

[34] Hobi M, Yegorova-Lee S, Chan CC-L, Zhao H, Jiang S, Tran D, et al. Strategies Australian junior doctors use to maintain their mental, physical and social well-being: a qualitative study. BMJ Open. 2022;12:e062631. 10.1136/bmjopen-2022-062631. 36581957 10.1136/bmjopen-2022-062631 PMC9438200

[35] Rimmer A. What should I eat on my night shift? BMJ. 2019;365:l2143. 10.1136/bmj.l2143. 31097433 10.1136/bmj.l2143

[36] Mutebi N. Health impacts of ultra-processed foods 2024. https://researchbriefings.files.parliament.uk/documents/POST-PB-0059/POST-PB-0059.pdf. Accessed 13 Aug 2025.

[37] Rico-Campà A, Martínez-González MA, Alvarez-Alvarez I, Mendonça RD, De La Fuente-Arrillaga C, Gómez-Donoso C, et al. Association between consumption of ultra-processed foods and all cause mortality: SUN prospective cohort study. BMJ. 2019;365:l1949. 10.1136/bmj.l1949. 31142450 10.1136/bmj.l1949 PMC6538973

[38] Lane MM, Gamage E, Du S, Ashtree DN, Mcguinness AJ, Gauci S, et al. Ultra-processed food exposure and adverse health outcomes: umbrella review of epidemiological meta-analyses. BMJ. 2024;384:e077310. 10.1136/bmj-2023-077310. 38418082 10.1136/bmj-2023-077310 PMC10899807

[39] Fertifa. The 'Period Gap': Why offices should have free sanitary products. 2025. https://www.fertifa.com/post/the-period-gap-why-offices-should-have-free-sanitary-products. Accessed 6 May 2025.

[40] Hopkin GP. NHS trusts should provide everyone with a locker. BMJ. 2022;379:o2404. 10.1136/bmj.o2404. 36216377 10.1136/bmj.o2404

[41] Yorks DM, Frothingham CA, Schuenke MD. Effects of group fitness classes on stress and quality of life of medical students. J Am Osteopath Assoc. 2017;117:e17–25. 10.7556/jaoa.2017.140. 29084328 10.7556/jaoa.2017.140

[42] Friedenberg R, Kalichman L, Ezra D, Wacht O, Alperovitch-Najenson D. Work-related musculoskeletal disorders and injuries among emergency medical technicians and paramedics: a comprehensive narrative review. Arch Environ Occup Health. 2022;77:9–17. 10.1080/19338244.2020.1832038. 33073742 10.1080/19338244.2020.1832038

[43] Verow P, Hargreaves C. Healthy workplace indicators: costing reasons for sickness absence within the UK National Health Service. Occup Med (Lond). 2000;50:251–7. 10.1093/occmed/50.4.251. 10912376 10.1093/occmed/50.4.251

[44] NHS England. Applications are invited for vacancies in Pre-Hospital Emergency Medicine 2025. https://heeoe.hee.nhs.uk/recruitment/pre-hospital-emergency-medicine-phem#:~:text=Details%20of%20the%20test%20and%20the%20certificate%20can,-%20if%20not%20attached%20to%20your%20PHEM%20application. Accessed 6 May 2025.

[45] Health Safety Executive. Personal protective equipment (PPE) at work regulations from 6 April 2022. 2022. https://www.hse.gov.uk/ppe/ppe-regulations-2022.htm. Accessed 6 May 2025.

[46] Vindrola-Padros C, Andrews L, Dowrick A, Djellouli N, Fillmore H, Bautista Gonzalez E, et al. Perceptions and experiences of healthcare workers during the COVID-19 pandemic in the UK. BMJ Open. 2020;10:e040503. 10.1136/bmjopen-2020-040503. 33154060 10.1136/bmjopen-2020-040503 PMC7646318

[47] Carbone R, Ferrari S, Callegarin S, Casotti F, Turina L, Aritoli G et al. Peer support between healthcare workers in hospital and out-of-hospital settings: a scoping review. Acta Biomed. 2022; 93:e2022308. 10.23750/abm.v93i5.13729. 10.23750/abm.v93i5.13729 PMC9686152 36300208

[48] Sinton D, Lewis G, Roland D. Excellence reporting (Greatix): creating a different paradigm in improving safety and quality. Emerg Med J. 2016;33:901–2. 10.1136/emermed-2016-206402.6.

[49] Mercy Murinye M. 257 Learning from excellence in healthcare practice: a positive event reporting system. BMJ Paediatrics Open. 2021;5:null. 10.1136/bmjpo-2021-RCPCH.144.

[50] Page J, Pearson S, Raghwan S. A qualitative evaluation of the hot debrief/follow-up initiative: implications of readily identifying positive outcomes in an Australian emergency department. J Nurs Manag. 2022;30:3589–98. 10.1111/jonm.13767. 35970197 10.1111/jonm.13767 PMC10087145

[51] North Bristol NHS Trust. North Bristol NHS Trust shortlisted for the 2022 HSJ awards. 2022. https://www.nbt.nhs.uk/about-us/news-media/latest-news/north-bristol-nhs-trust-shortlisted-2022-hsj-awards. Accessed 8 May 2025.

[52] Walker CA, Mcgregor L, Taylor C, Robinson S. STOP5: a hot debrief model for resuscitation cases in the emergency department. Clin Exp Emerg Med. 2020;7:259–66. 10.15441/ceem.19.086. 33440103 10.15441/ceem.19.086 PMC7808839

[53] Carenzo L, Baker C, Jones S, Hurst T. A framework for case-based learning in prehospital medicine: the London’s Air Ambulance experience. Air Med J. 2022;41:521–5. 10.1016/j.amj.2022.09.005. 36494166 10.1016/j.amj.2022.09.005

[54] UK Search and Rescue. Guiding principles for emergency response volutneer wellbeing. 2023. https://assets.publishing.service.gov.uk/media/658ecce180a3bb000e9d05f2/Guiding_principles_-Final_with_updated_logos.pdf. Accessed 8 May 2025.

[55] Institute for Pre-Hospital Emergency Medicine. Introduction. 2025. https://phem.org.uk/about. Accessed 8 May 2025.

[56] General Medical Council. Continuing professional development: Guidance for all doctors. 2012. https://www.gmc-uk.org/-/media/documents/cpd-guidance-for-all-doctors-0316_pdf-56438625.pdf. Accessed 8 May 2025.

[57] FPHC. Descriptors. 2025. https://fphc.rcsed.ac.uk/media/2911/phem-competency-descriptors-and-framework.pdf. Accessed 8 May 2025.

[58] Yusoff MS. Associations of pass-fail outcomes with psychological health of first-year medical students in a Malaysian medical school. Sultan Qaboos Univ Med J. 2013;13:107–14. 10.12816/0003203. 23573390 10.18295/2075-0528.1437 PMC3616775

[59] Faculty of Intensive Care Medicine. Resources for candidates. 2025. https://www.ficm.ac.uk/trainingexamsexaminations/resources-for-candidates. Accessed 4 July 2025.

[60] Williams R, Kemp, V., Stokes, S., Lockey, D. Peer Support: An introductory or briefing document. 2020. https://fphc.rcsed.ac.uk/media/2841/peer-support.pdf. Accessed 4 July 2025.

[61] Intensive Care Society. The Intensive Care Society strategic framework for peer support. 2020. https://ics.ac.uk/resource/peer-support.html#:~:text=Please%20refer%20to%20our%20Strategic%20Framework%20for%20Peer,its%20principles%2C%20values%20and%20structure%20for%20safe%20delivery. Accessed 13 Aug 2025.

[62] Maddock M, Kemp V and Williams R. Case Study 4: Delivering Peer Support. Major Incidents, Pandemics and Mental Health: The Psychosocial Aspects of Health Emergencies, Incidents, Disasters and Disease Outbreaks. 2024;360–4. https://www.cambridge.org/core/product/4835115E5659C783CD7EB38D3BE357C4.

[63] General Medical Council. Revalidation. 2025. https://www.gmc-uk.org/registration-and-licensing/managing-your-registration/revalidation. Accessed 8 May 2025.

